# Identification of signatures associated with microsatellite instability and immune characteristics to predict the prognostic risk of colon cancer

**DOI:** 10.1515/med-2024-1056

**Published:** 2024-12-20

**Authors:** Sihan Bo, Yong You, Yongwei Wang, Yan Zhang, Bing Bai, Tao Jiang, Yaxian Gao

**Affiliations:** Department of Immunology, Basic Medical Institute, Chengde Medical College, Chengde 067000, Hebei, China; Department of Anatomy, Basic Medical Institute, Chengde Medical College, Chengde 067000, Hebei, China

**Keywords:** colon cancer, microsatellite instability, immune microenvironment, prognostic model, weighted gene co-expression network analysis

## Abstract

**Background:**

Microsatellite instability (MSI) significantly impacts treatment response and outcomes in colon cancer; however, its underlying molecular mechanisms remain unclear. This study aimed to identify prognostic biomarkers by comparing MSI and microsatellite stability (MSS).

**Methods:**

Data from the GSE39582 dataset downloaded from the Gene Expression Omnibus database were analyzed for differentially expressed genes (DEGs) and immune cell infiltration between MSI and MSS. Then, weighted gene co-expression network analysis (WGCNA) was utilized to identify the key modules, and the modules related to immune infiltration phenotypes were considered as the immune-related gene modules, followed by enrichment analysis of immune-related module genes. Prognostic signatures were derived using Cox regression, and their correlation with immune features and clinical features was assessed, followed by a nomogram construction.

**Results:**

A total of 857 DEGs and 14 differential immune cell infiltration between MSI and MSS were obtained. Then, WGCNA identified two immune-related modules comprising 356 genes, namely MEturquoise and MEbrown. Eight signature genes were identified, namely *PLK2*, *VSIG4*, *LY75*, *GZMB*, *GAS1*, *LIPG*, *ANG*, and *AMACR*, followed by prognostic model construction. Both training and validation cohorts revealed that these eight signature genes have prognostic value, and the prognostic model showed superior predictive performance for colon cancer prognosis and distinguished the clinical characteristics of colon cancer patients. Notably, *VSIG4* among the signature genes correlated significantly with immune infiltration, human leukocyte antigen expression, and immune pathway enrichment. Finally, the constructed nomogram model could significantly predict the prognosis of colorectal cancer.

**Conclusion:**

This study identifies eight prognostic signature genes associated with MSI and immune infiltration in colon cancer, suggesting their potential for predicting prognostic risk.

## 1 Introduction

Colon cancer is formed by cancerous epithelial cells of the colon in the gastrointestinal tract and is the fourth most common lethal cancer, causing approximately 800,000 deaths worldwide each year [1,2,3]. Bacterial infections, an unhealthy lifestyle, such as high-fat diets, high alcohol consumption, and genetic factors are believed to be the main causes for the growth and invasion of heterogeneous colon cancer cells [4]. Current basic treatment options include endoscopic and surgical resection, systemic adjuvant chemotherapy, radiotherapy, targeted therapy, and immunotherapy [5]. However, the estimated 5-year survival rate for advanced metastatic colon cancer is still less than 15%, possibly owing to a lack of response to treatment [6,7]. Specific genetic alterations, including chromosomal abnormalities, epigenetic regulation, mutations in *KRAS*, *BRAF*, *PI3K*, *p53*, and other genes, are involved in malignant transformation, infiltrative formation, and distal metastasis of colon cancer [8,9]. Therefore, mining reliable genetic and molecular regulation biomarkers is necessary to predict the response to treatment and improve the clinical outcomes of patients.

Microsatellites are short tandem repeats of DNA sequences, the repetitive structures of which are distributed across the coding and non-coding regions of the genome and often lead to mismatch repair [10]. Mismatch repair deficiency, which manifests as high microsatellite instability (MSI), promotes tumorigenesis in 15% of colon cancers via a unique mechanism [11,12,13]. High MSI levels lead to molecular changes, such as amassing of somatic mutations, high levels of tumor mutation burden, increased neoantigen expression, and enhanced lymphocytic infiltration [14]. In colon cancer, MSI and microsatellite stability (MSS) phenotypes exhibit certain differences in clinical and pathological features, including mucinous tissue differentiation, lymphocytic infiltration level, tumorigenesis location, and lymph node metastasis [15]. MSI is considered a predictor of immunotherapy sensitivity because it is more sensitive to immunosuppressive checkpoint inhibitors than to 5-fluorouracil [16]. Studies have also reported that patients with early-stage colorectal cancer with MSI usually have a favorable prognosis because they are more likely to respond to immunotherapy [17]. However, up to 50% of patients with MSI in advanced colon cancer continue to progress after PD-1 blockade [18]. Therefore, even if the microsatellite status of colon cancer has clinical significance in predicting prognosis and guiding treatment, the exploration of the molecular mechanisms of MSI and MSS that may affect prognosis is still indispensable.

To date, there is still a lack of relevant studies to systematically explore the molecular and immunomodulatory mechanisms of MSI and MSS in colon cancer prognosis. Therefore, this study compared their expression profiles based on data from public databases and identified immune-related prognostic signatures using weighted gene co-expression network analysis (WGCNA). The prognostic model and nomogram predictor constructed based on these signatures can identify the prognostic risk of colon cancer. In addition, we explored the characteristics of the immune microenvironment in MSI and MSS, as well as between patients with high and low prognostic risks, to further elucidate the possible involvement of these key genes in immune regulatory mechanisms. Our findings may further explain the involvement of MSI in tumor metastasis and recurrence, and provide new prospects for improving prognosis and clinical outcomes in colon cancer.

## 2 Materials and methods

### 2.1 Data acquisition and filtering

The GSE39582 and GSE17536 RNA microarray data were acquired from the Gene Expression Omnibus (GEO) database. After excluding 17 non-cancer samples and 9 colon cancer samples with missing survival data, 510 colon cancer samples in the GSE39582 dataset were included in this study. Also, 177 colon cancer samples were contained in the GSE17536 dataset. In addition, the expression profiles and clinical information (including overall survival [OS], OS time, age, sex, pathologic T, N, and M TNM stages, adjuvant chemotherapy, and microsatellite status) were incorporated in this study. In this study, the GSE39582 and GSE17536 datasets served as the training and validation cohorts, respectively. Furthermore, the colon cancer samples in the GSE39582 dataset were divided into MSI and MSS groups: 71 samples in the MSI group, and 439 samples in the MSS group. For data preprocessing, the expression data were first normalized by log2, according to the probe values of the matrix. The second step was gene annotation. The platform annotation file GPL570 provided in the GEO database was used to transform the probe and gene symbols, followed by the merging of all probes using the mean values. The SVA package [19] was employed to preprocess and remove batch effects.

### 2.2 Screening MSI and MSS differentially expressed genes (DEGs)

The limma (3.9.19) package [20] in R was utilized for background correction, normalization between arrays, and DEG identification between MSI and MSS groups. Benjamini and Hochberg's procedure was used to correct the *p* values. An adj. *p* value <0.05 and |log fold change (FC)| > 0.585 were set to identify statistical significance, which was further visualized with a volcano plot generated with the ggpubr (v. 0.4.0) package.

### 2.3 Comparison of MSI and MSS immune infiltration scores

To probe the relationship between microsatellite status and the immune microenvironment, the deconvolution method in the CIBERSORT (4.0.2) R package [21] was used to determine the ratio of the 22 types of immune cells in each sample. Based on the “LM22 leukocyte gene matrix set,” the deconvolution method was used to analyze the multiple immune cell types in all samples. Then, the Mann–Whitney *U* test was applied to analyze the difference in immune infiltration scores between the MSI and MSS groups, and the statistical significance was set at *p* < 0.05. The ggplot package in R was used to create box plots.

### 2.4 WGCNA for screening immune-related modules

To observe the correlations between DEGs in MSI and MSS, a WGCNA R package [22] was used to explore the differential gene module network. For quality control, genes with a top 75% of median absolute deviation (MAD) >0.01 were screened. The sampleTree function was then used to perform hclust clustering on the sample population to remove outlying samples. Based on the above quality control process, a co-expression network of DEGs between MSI and MSS was constructed. Subsequently, the pickSoftThreshold function of the R WGCNA package (v. 1.71) was employed to calculate the appropriate power (*R*-square >0.85) for network construction (maxBlockSize = 5,000). The formulas for calculating edge attributes were:

undirected network: \[\text{Abs}{(\text{Cor}(\text{genex,}\text{geney}))}^{\text{power}}]\] ;

directed network: \[{\left(,1+\frac{\text{Cor}(\text{genex,}\text{geney})}{2}\right)}^{\text{power}}]\] ;

sign hybrid: \[\text{Cor}{(\text{genex,}\text{geney})}^{\text{power}}\hspace{.25em}\text{if}\hspace{.25em}\text{Cor}> 0\hspace{.25em}\text{else}\hspace{.25em}0]\] .

The plotDendro and Colors function was used to create the hierarchical clustering of gene modules.

With the identification of gene modules, the relationship between gene modules and immune infiltration phenotypes that differed significantly between MSS and MSI was analyzed using Spearman’s correlation analysis. The immune-related modules were selected at a *p*-value < 0.05 and |Coeff| >0.2.

### 2.5 Enrichment analysis of immune-related module genes

For the genes in the immune-related modules obtained above, the R clusterProfiler package [23] was used to convert gene IDs to *Homo sapiens* Entrez gene IDs and perform Gene Ontology (GO) functional and Kyoto Encyclopedia of Genes and Genomes (KEGG) pathway enrichment analyses. Enrichment terms with *p* < 0.05 and enrichment factor >1.5 were identified as statistically significant, and the top 5 enriched terms were visualized.

### 2.6 Construction and verification of an immune-related predictive model

For genes in the immune-related modules, univariate Cox regression in the survival package [24] (v. 4.1-3) was applied to select genes that were significantly associated with OS with a cutoff value of *p* < 0.01. Stepwise Cox regression was performed using the survival package (v 4.1-3) to construct a prognostic model based on the obtained prognostic genes according to the following formula:

\[{\text{Risk score}}_{\text{sample}}=\mathop{\sum }\limits_{1}^{n}{\text{Coef}}_{i}\times {x}_{i}.]\]

Here, \[{\text{Coef}}_{i}]\] indicates the regression coefficient and \[{x}_{i}]\] represents gene expression. The samples were then clustered into high- and low-risk groups based on the medium-risk score. To explore the difference in OS between the two groups, the Kaplan–Meier (KM) algorithm provided in the R survival package (v. 3.3-1) was adopted for survival estimation. The R pROC (v. 1.18.0) package was used to generate receiver operating characteristic (ROC) curves and their area under the curve (AUC) to identify the predictive potential of the model in terms of sensitivity and specificity. Samples from the GSE17536 dataset were enrolled for prognostic model validation; the difference in survival probability between patients with high and low prognostic risks was estimated by calculating their risk scores to further verify the reliability of prognostic signatures for survival determination.

### 2.7 Clinical feature analysis among risk groups

According to the risk group obtained above, the chi-squared test and *t*-test were utilized to analyze differences in clinical phenotypes between the high- and low-risk groups where appropriate, including age, sex, pathologic TNM stages, adjuvant chemotherapy, and microsatellite status.

### 2.8 Immune microenvironment assessment among risk groups

To explore the differences in the activity of immune-related cells and risk score grouping, first, the fraction of 22 immune cells was calculated using the CIBERSORT algorithm, followed by a comparison between the high- and low-risk groups using the Wilcox test. Then, the Spearman correlation analysis between the gene expression matrix of the prognostic model and significantly different immune infiltrating cells was carried out with a cutoff value of *p* < 0.05 and |Coeff| > 0.2. Second, the R ESTIMATE package [25] (v. 1.0.13) was used to calculate stromal, immune, and ESTIMATE scores as well as tumor purity, and the differences between groups were compared using the Wilcox test with a threshold of *p* < 0.05. Third, the expression of human leukocyte antigen (HLA) family genes was extracted and compared between high- and low-risk groups using the Wilcox test with a threshold of *p* < 0.05. Fourth, the enrichment scores of 17 immune pathways, which were obtained from the ImmPort database, were assessed through gene set variation analysis (GSVA) using the GSVA package (v. 1.36.3), followed by the comparison of differences between the high- and low-risk groups using the Wilcox test with a threshold of *p* <0.05. Finally, the ESTIMATE scores, HLA genes, and GSVA pathways that differed significantly between the risk groups (*p* in the Wilcox test < 0.05) were analyzed for Spearman correlation with prognostic signatures (*p* < 0.05, |Coeff| > 0.2).

### 2.9 Screening of independent prognostic risk factors to construct a nomogram predictor

Univariate Cox proportional hazard regression was performed on nine indicators, including clinical indexes (age, chemotherapy adjuvant, sex, M, N, risk score, and TNM stage) and risk score groups, and the indicators with prognostic significance were included in the multivariate Cox regression to further identify independent prognostic risk factors with a cutoff value of *p* < 0.05. Clinical indicators with prognostic significance were also used to generate a nomogram model for predicting survival using the nomogram function. Furthermore, we calculated the consistency index (c-index) and plotted the ROC curve, as well as the calibration curve using the calibrate function, to validate the predictive efficiency of the nomogram model.

### 2.10 Statistical analysis

*R* software (v. 4.2.1; https://www.r-project.org/) and various R packages were utilized for all statistical and data analyses. Wilcox test and chi-squared test were used for pairwise comparisons. Comparisons of OS were conducted using a survival package with Kaplan–Meier curves. The results of the analysis were made statistically significant at *p* < 0.05.

## 3 Results

### 3.1 Differences in gene expression and immune infiltration between MSI and MSS

**Figure 1:**
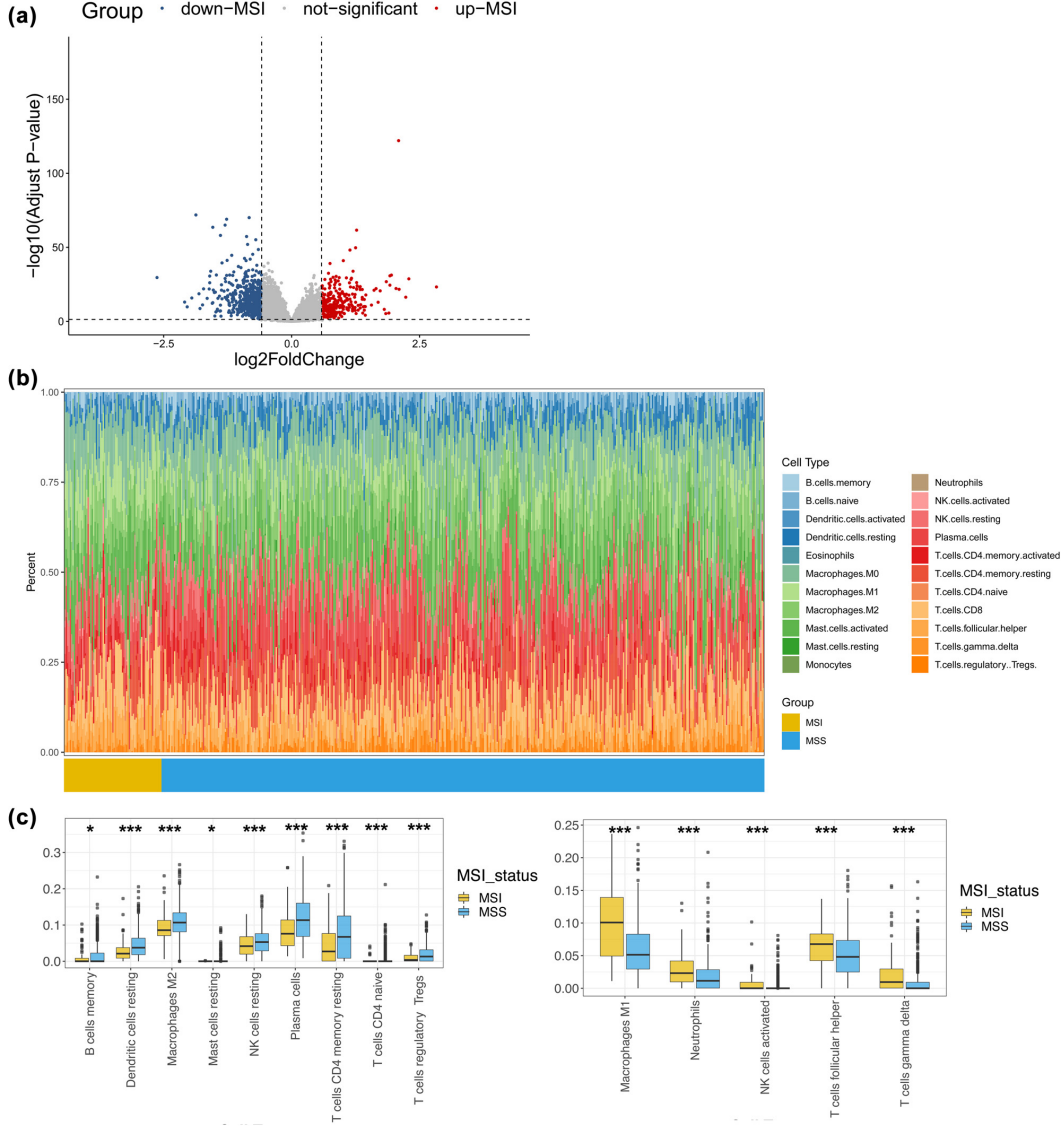
Differential analysis of gene expression profiles and immune cell infiltration between patients with MSI and MSS. (a) The volcano plot shows 857 DEGs between MSI and MSS screened with an adj. *p* < 0.05 and |log fold change (FC)| > 0.585. (b) The CIBERSORT algorithm was used to calculate the ratio of 22 types of immune cells in 510 samples from the GSE39582 dataset. (c) Box plots depict 14 types of immune cells with significant differences in infiltration between MSI and MSS. *p < 0.05, **p < 0.01, and ***p < 0.001. *p* < 0.05 indicates statistical significance.

A total of 857 DEGs were selected by comparing the MSI and MSS expression profiles (Figure 1a). Furthermore, immune cell fractions from 510 samples in the GSE39582 dataset were analyzed using the CIBERSORT algorithm (Figure 1b). Differential analysis indicated that 14 types of immune cells differed significantly in infiltration abundance between the MSI and MSS groups. Nine of these were highly infiltrated in the MSS group, whereas five were markedly increased in the MSI group (all *p* < 0.05; Figure 1c).

### 3.2 Screening of immune-related modules using WGCNA

**Figure 2:**
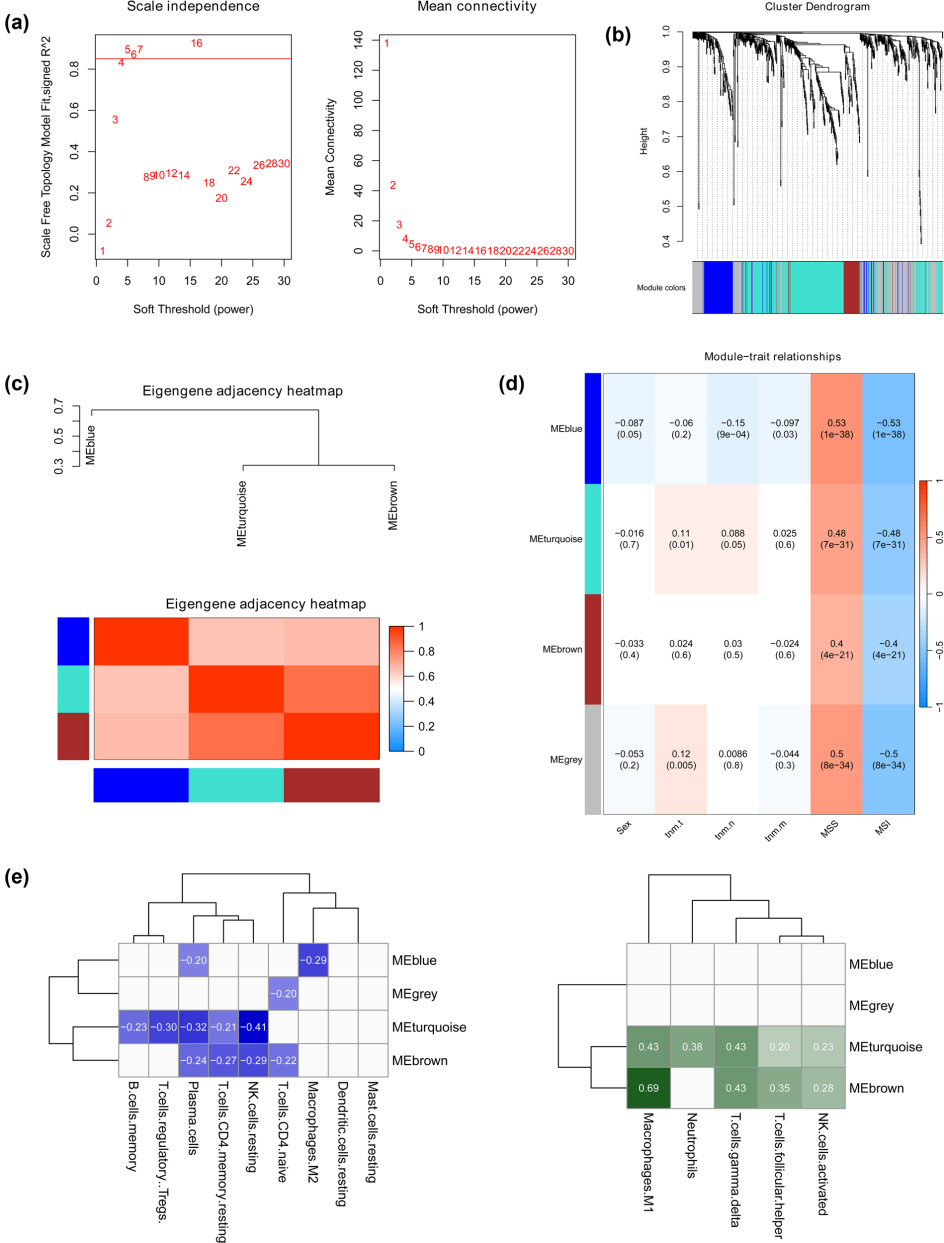
WGCNA and screening of immune-related modules. (a) The soft threshold was determined according to an *R*-square = 0.85. (b) Four clustering modules, MEblue, MEbrown, MEgrey, and MEturquoise, were identified using WGCNA. (c) MEblue, MEbrown, and MEturquoise modules are significantly correlated. (d) Correlation between four clustering modules and phenotypes. (e) Immune-related modules were identified by evaluating Spearman’s correlation between the 4 key modules and 14 immune cells.

A total of 857 DEGs were incorporated into the WGCNA. After excluding the genes with the top 75% of MAD and eliminating outlier samples, the expression matrix of 642 genes from 510 samples was finally included for further analysis. Furthermore, *R*-square = 0.85 was set to determine the soft threshold for co-expression network analysis (Figure 2a), and WGCNA suggested four clustering modules, MEblue, MEbrown, MEgrey, and MEturquoise (Figure 2b), which contained 100, 51, 186, and 305 genes, respectively. The results of the correlation analysis between modules indicated that there was a significant positive correlation between MEblue, MEbrown, and MEturquoise (Figure 2c). Also, a correlation between four clustering modules and phenotypes was explored, and the results showed that MEblue, MEbrown, MEgrey, and MEturquoise were significantly correlated with MSI and MSS (Figure 2d). To further identify immune-related modules, Spearman’s correlation analysis was performed on the 4 key modules and 14 types of immune cells with significant differences in infiltration between MSI and MSS. With *p* < 0.05, |Coeff| > 0.2, MEturquoise, and MEbrown were identified as immune-related modules (Figure 2e).

### 3.3 Enrichment analyses of immune-related module genes

**Figure 3:**
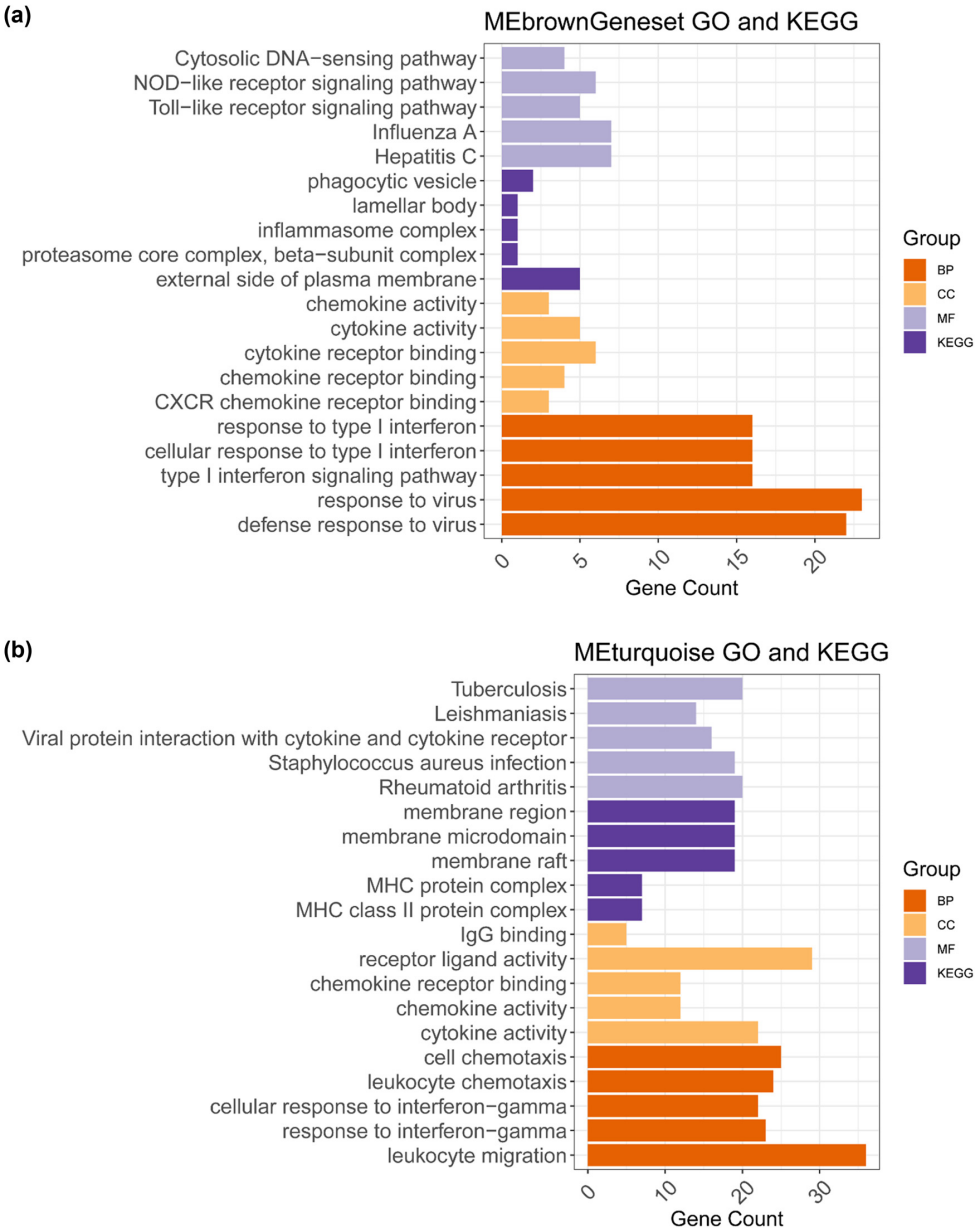
Gene ontology (GO) functions and KEGG pathway enrichment analyses of genes in immune-related modules. (a) Top five enriched GO and KEGG terms for the 51 genes in the MEbrown module. (b) Top five enriched GO and KEGG terms for the 305 genes in the MEturquoise module.

The MEturquoise and MEbrown modules contained gene sets with 305 and 51 genes, respectively, for which GO and KEGG enrichment analyses were performed. Among them, 51 genes of the MEbrown module were mainly enriched in GO functions in response to viruses, cytokine receptor binding, influenza A, and hepatitis C, as well as in KEGG pathways on the external side of the plasma membrane (Figure 3a). Furthermore, 305 genes in the MEturquoise module were mainly involved in GO functions of leukocyte migration, receptor-ligand activity, tuberculosis, *Staphylococcus aureus* infection, and rheumatoid arthritis, as well as in KEGG pathways of the membrane region, microdomain, and raft (Figure 3b).

### 3.4 Evaluation of the predictive model based on immune-related module genes

**Figure 4:**
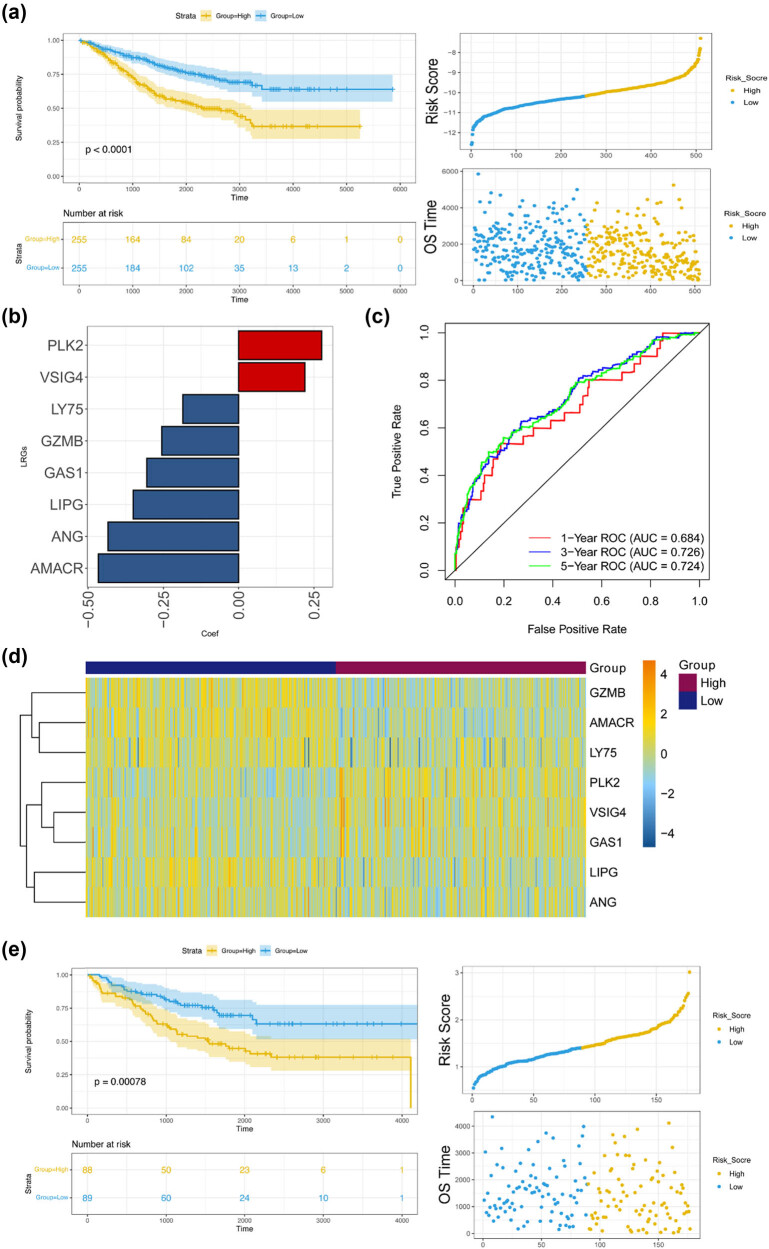
Prognostic model construction and validation. Based on the eight immune-related prognostic signatures, the prognostic model was constructed and validated on the GSE39582 and GSE17536 datasets, respectively. (a) The KM curve based on the training set shows the survival differences between the high- and low-risk groups. (b) Regression coefficients of the eight prognostic signatures in the GSE39582 dataset. (c) 1-, 3-, and 5-year ROC curves of the prognostic model. (d) Expression distribution of the eight signatures in high- and low-risk samples from the GSE39582 dataset. (e) KM survival validation of the prognostic model using the GSE17536 dataset. *p* < 0.05 indicates statistical significance.

**Figure A1:**
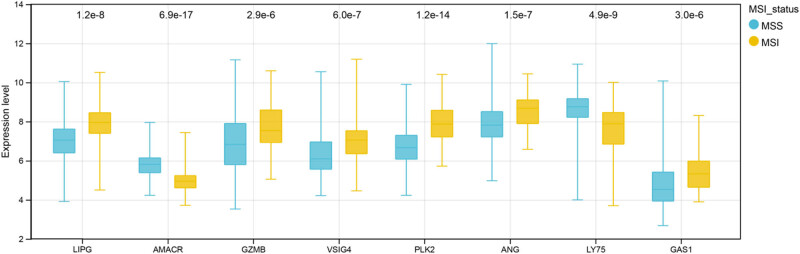
Expression differences between MSI and MSS of eight prognostic signatures.

By integrating the expression data and survival information, 40 genes significantly associated with prognosis were selected from 356 immune-related module genes. Stepwise Cox regression analysis was conducted on these 40 prognostic genes, and eight signature genes were further identified (namely, *PLK2*, *VSIG4*, *LY75*, *GZMB*, *GAS1*, *LIPG*, *ANG*, and *AMACR*) for prognostic model construction. After calculating the risk score using the expression levels and regression coefficients of the prognostic signatures, samples in the GSE39582 dataset were grouped into high- and low-risk groups, among which a significant survival difference was observed (*p* < 0.0001), and patients in the high-risk group were prone to adverse prognosis (Figure 4a). The regression coefficients of the eight prognostic signatures in the GSE39582 dataset are shown in Figure 4b. The AUCs of the 1-, 3-, and 5-year ROC curves were 0.684, 0.726, and 0.724, respectively, suggesting that the model has a high prediction accuracy (Figure 4c). The expression distribution of the eight signature genes in all the samples of the training set is depicted in Figure 4d; *PLK2*, *VSIG4*, and *GAS1* tended to be highly expressed in the low-risk group, whereas the other five genes were prone to being downregulated. These eight genes were also confirmed to have significant differences in expression between MSI and MSS (Figure A1). Furthermore, the risk scores of the GSE17536 samples were estimated to validate the reliability and stability of the model. The KM curve confirmed that patients with a high prognostic risk had a more adverse survival prognosis (Figure 4e). These results suggest that the eight signature genes have prognostic values and that the prognostic model shows a superior predictive performance for colon cancer prognosis.

### 3.5 Differences in clinical features among risk groups

Clinical characteristics were compared between patients with high and low prognostic risk using the *t*-test and chi-square test, where appropriate. The results indicate that three clinical indices, including TNM stage, pathological T, and pathological N, varied substantially between the two groups, and patients with high prognostic risk were more distributed in the malignant clinical phenotypes, such as the advanced TNM stage (all *p* < 0.05; Table 1). These results also suggest that prognostic signatures related to microsatellite status can substantially distinguish the clinical characteristics of colon cancer patients.

**Table 1:** Clinical characteristics of patients in high- and low-risk groups

Characteristics	Low (*N* = 255)	High (*N* = 255)	Total (*N* = 510)	*p*
**Sex**				0.13
Female	103(20.20%)	121(23.73%)	224(43.92%)	
Male	152(29.80%)	134(26.27%)	286(56.08%)	
**Age**				0.15
Mean ± SD	66.44 ± 12.01	67.44 ± 14.56	66.94 ± 13.34	
Median[min-max]	67.50[31.70,96.00]	69.50[22.00,97.00]	68.00[22.00,97.00]	
**TNM stage**				**6.40 × 10** ^**−3**^
Stage 1	24(4.74%)	8(1.58%)	32(6.32%)	
Stage 2	113(22.33%)	104(20.55%)	217(42.89%)	
Stage 3	93(18.38%)	107(21.15%)	200(39.53%)	
Stage 4	22(4.35%)	35(6.92%)	57(11.26%)	
**Pathological T**				**1.00 × 10** ^**−3**^
T1	7(1.44%)	4(0.82%)	11(2.26%)	
T2	33(6.79%)	11(2.26%)	44(9.05%)	
T3	164(33.74%)	166(34.16%)	330(67.90%)	
T4	40(8.23%)	61(12.55%)	101(20.78%)	
**Pathological N**				**0.04**
N0	137(28.31%)	115(23.76%)	252(52.07%)	
N1	67(13.84%)	62(12.81%)	129(26.65%)	
N2	36(7.44%)	61(12.60%)	97(20.04%)	
N3	3(0.62%)	3(0.62%)	6(1.24%)	
**Pathological M**				0.08
M0	224(45.90%)	207(42.42%)	431(88.32%)	
M1	22(4.51%)	35(7.17%)	57(11.68%)	
**Chemotherapy adjuvant**				0.58
No	141(28.54%)	135(27.33%)	276(55.87%)	
Yes	105(21.26%)	113(22.87%)	218(44.13%)	
**Microsatellite status**				0.31
MSI	31(6.08%)	40(7.84%)	71(13.92%)	
MSS	224(43.92%)	215(42.16%)	439(86.08%)	

SD: standard deviation; TNM: tumor, node, metastasis; MSI: microsatellite instability; MSS: microsatellite stability.

Boldface *p* < 0.05 indicates statistical significance.

### 3.6 Identification of immune features in the two risk groups

**Figure 5:**
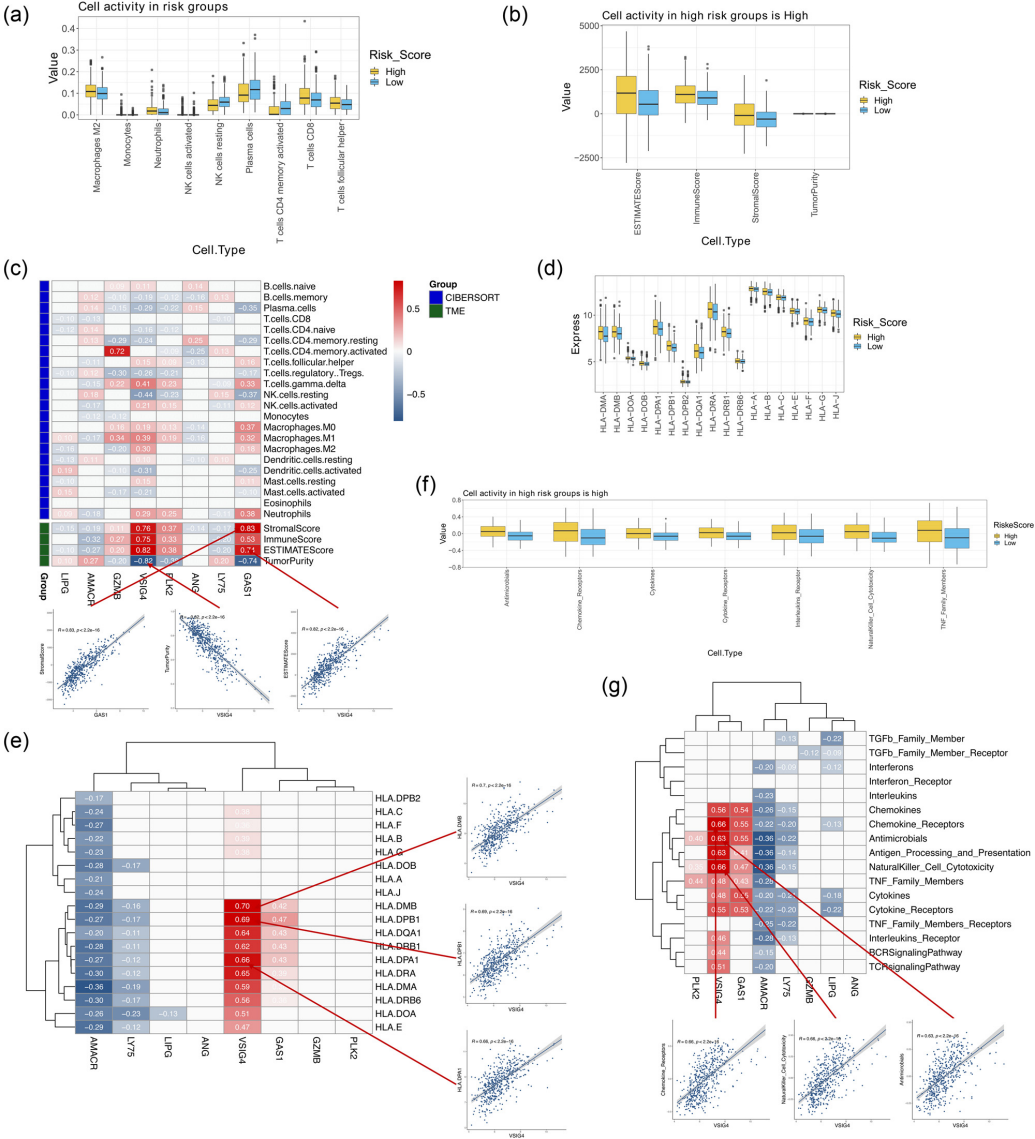
Differences in immune characteristics between high- and low-risk groups and their correlation with the eight prognostic signatures related to microsatellite status. (a) Nine immune cell types with significant differences in infiltration between high- and low-risk groups. (b) Box plots showing significant differences in ImmuneScore and TumorPurity between the two groups. (c) Correlations between the expression of the eight signatures and immune cell infiltration, stromal, immune, and ESTIMATE scores, as well as tumor purity. (d) Nine HLA genes with significant differences between the two groups. (e) Spearman’s correlation of expression levels between HLA family genes and the eight prognostic signatures. (f) Seven of the 17 immune pathways showed significant enrichment score differences between the two groups. (g) Spearman’s correlation between 17 GSVA pathways and the eight model signature genes. *p* < 0.05 indicates statistical significance.

Using the CIBERSORT algorithm, nine types of immune cells were found to have significant differences in their infiltration levels between the two groups (all *p* < 0.05; Figure 5a). Furthermore, the stromal, immune, and ESTIMATE scores, as well as tumor purity, showed significant differences between high- and low-risk groups (Figure 5b). Correlation analysis indicated the strongest positive correlation between VSIG4 and StromalScore, and the strongest negative correlation between VSIG4 and TumorPurity (Figure 5c). In addition, the expression of eight HLA genes varied markedly between the two groups (all *p* < 0.05; Figure 5d), and *VSIG4* and *HLA-DMB* shared the highest positive relationship in expression levels (Figure 5e). Using the GSVA algorithm, we also found significant differences in seven immune pathways between the two groups (all *p* < 0.05; Figure 5f), in which *VSIG4* had the strongest positive correlation with natural killer cell cytotoxicity and chemokine receptors (Figure 5g).

### 3.7 Prognostic independence analysis and nomogram prediction model construction

**Figure 6:**
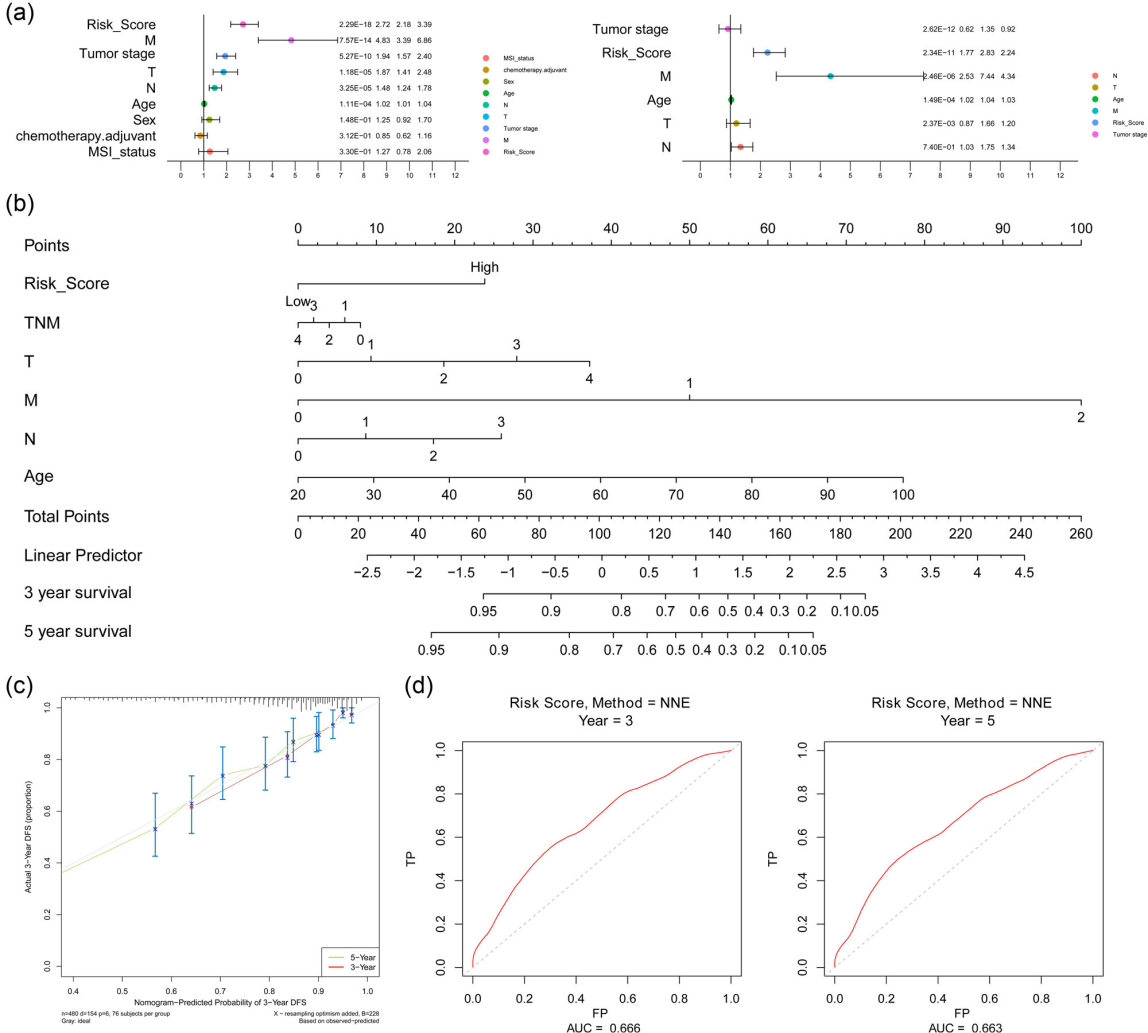
Identification of independent prognostic factors and construction of a nomogram predictor. (a) The pathological T, N, M, and risk scores were defined as independent prognostic factors using univariate and multivariate Cox regression analyses. (b) Six prognostic factors were included to construct a nomogram. (c) Calibration curves showing a high fit between the predicted and actual values for the 3- and 5-year survival probabilities. (d) 3- and 5-year ROC curves were used in the nomogram to evaluate the predictive performance of the model.

Univariate Cox regression analysis revealed that pathological T, N, and M stages, as well as age, tumor stage, and risk score, had prognostic significance (Figure 6a). These factors were further incorporated into the multivariate Cox regression analysis, in which pathological T, N, M, and risk scores were identified with prognostic independence (Figure 6a). Then, six factors with prognostic significance were included to build a nomogram prediction model with a c-index of 0.749 (Figure 6b), indicating high uniformity between the predicted probability and actual value. Furthermore, the plotted calibration curves showed a high fit between the predicted and actual values for the 3- and 5-year survival probabilities (Figure 6c). The AUCs of the 3- and 5-year ROC curves were 0.666 and 0.663, respectively (Figure 6d), suggesting that the nomogram model can significantly predict the prognosis of colorectal cancer.

## 4 Discussion

By comparing the expression profiles of 510 patients with colon cancer, 857 DEGs between MSI and MSS and 14 immune cell types with significant infiltration differences were identified in this study. We found that MSI-high colon cancer has more immune cell infiltration, higher expression of immune-related genes, and higher immunogenicity compared to MSS or MSI-low cases [26]. Our study identified five types of immune cells that were highly infiltrated in MSI, whereas the other nine were highly infiltrated in MSS. It has been reported that MSI-high colorectal cancer with a large number of *Fusobacterium nucleatum* is associated with increased infiltration of macrophages [27]. Furthermore, the ratio of M2-polarized macrophages in tumor centers is increased in MSI-high colorectal cancer subsets [28]. CD4+ and CD8+ T cell levels are higher in patients with MSI-high colon cancer than in those with MMS [29]. However, we found contrasting findings in the infiltration levels of M2 macrophages and CD4+ T cells, probably because the MSI levels were not further stratified in this study, and MSI-low tended to converge with the MSS phenotype.

In this study, the eight prognostic signatures related to immunophenotypes (*PLK2*, *VSIG4*, *LY75*, *GZMB*, *GAS1*, *LIPG*, *ANG*, and *AMACR*) were further screened among the DEGs between MSI and MSS with the WGCNA. These genes had different expression distributions between the prognostic risk groups, with *PLK2*, *VSIG4*, and *GAS1* being highly expressed in the high-risk group and possibly serving as prognostic risk factors. Prognostic models constructed based on the expression of these eight genes can stratify and identify the prognostic risk of colon cancer, which provide new possibilities for personalized treatment. Importantly, the novel constructed a prognostic risk model with independent prognostic value to help clinicians provide a meaningful reference for the prognosis and treatment of colon cancer patients. Among them, *PLK2* was found to induce the growth and proliferation of colorectal cancer cells, whereas TIG1 prevents this process [30]. VSIG4 positivity in patients with advanced gastric cancer is associated with an adverse prognosis, but the relationship between VSIG4 expression and colon cancer survival has not been observed [31]. *In vivo* mouse-based experiments have also confirmed higher mRNA levels of GAS1 in tumor tissues than those in normal tissues [32]. Additionally, the expression of AMACR in colorectal adenoma and carcinoma tissues is higher than that in non-tumoral epithelial tissues, suggesting its potential role in tumor formation [33]. Moreover, AMACR is positively expressed in 81.7% of patients with colorectal cancer and is significantly correlated with several clinical features but not with patient outcomes [34]. The role of the other four genes in colon cancer has not been reported, but these previous findings can support our results to a certain extent and indicate that the candidate genes have important roles in colon carcinogenesis, which might provide potential therapeutic targets for colon cancer. However, their potential as prognostic markers still needs to be validated in large sample cohorts. In addition, a nomogram combining this model with conventional clinical parameters like the TNM stage showed significantly improved performance, especially in predicting short-term survival (3-year or 5-year), indicating a more accurate reflection of the great heterogeneity of colon cancer.

Furthermore, immune cell populations are different between high- and low-risk patients, as well as the stromal, immune, and ESTIMATE scores. Thus, risk scores could be used to evaluate immune infiltration in colon cancer patients, which may be useful for immune-targeted tumor therapy. Notably, the high-risk group is more associated with immune cell infiltration and high stromal, immune, and ESTIMATE scores, and therefore the patients in high-risk group are more likely to benefit from immunotherapy. To further assess the relationship between the eight prognostic genes and immune characteristics, we conducted Pearson’s correlation analysis, which suggested a significant positive correlation between *VSIG4* and tumor cell infiltration, immune score, HLA gene expression, and GSVA immune pathways. Furthermore, higher expression of *VSIG4* in MSI was observed in this study. A relevant study pointed out that MSI is associated with the production and accumulation of cellular mutations, which may lead to significant neoantigen exposure and facilitate the initiation of a more efficient anti-tumor immune response [35]. Thus, *VSIG4* may be involved in the activation of these immune responses and lead to positive regulation of *VSIG4* in relation to the majority of tumor immune features. *VSIG4* is also known as Z39Ig, and its protein signal significantly induces the expression of HLA-DR on the surface of immune cells, thereby activating the immune response [36,37]. Notably, relationships between *VSIG4*, *HLA-DRA*, and *HLA-DRB6* were observed in this study, indicating that *VSIG4* may mediate the expression of HLA-DR family genes through antigen presentation and be involved in the immune activation mechanism of MSI colon cancer. In addition, *VSIG4* and *HLA-E* were found to be co-expressed in M2 macrophages in renal clear cell carcinoma, enriched in neutrophil activation, and involved in immune responses [38]. The present study confirmed that *VSIG4* is positively correlated with *HLA-E* expression (*r* = 0.47), ratio of M2 macrophages (*r* = 0.30), and neutrophil infiltration (*r* = 0.29). Additionally, we found that *AMACR* and *LY75* were significantly under-expressed in MSI, up-regulated in the low-risk group, and negatively correlated with the expression of most HLA family genes and immune pathways. Therefore, we speculate that *AMACR* and *LY75* may inhibit tumor mutation burden in colon cancer and lead to decreased MSI levels, while the suppressed anti-tumor immune response will further reduce the risk of colon cancer recurrence and proliferation. However, the immune regulatory mechanisms involved in these key genes require further investigation.

Studies based on public datasets are often limited by the availability of clinical information. For example, the two datasets included in this study did not allow for a comparison between MSI-low and MSI-high groups owing to a lack of information on MSI levels. In addition, the vast majority of colon cancer datasets lack information on microsatellite status, resulting in fewer datasets being available for validation. Therefore, in a follow-up study, we will consider collecting solid tumor samples, including sufficiently detailed clinical information, to explore whether prognostic signatures can be associated with differentiating prognostic risk between MSS, MSI-low, and MSI-high colon cancers and to investigate the potential relationship between the expression of key genes and microsatellite status. In addition, because of the individual differences and other confounding factors, the results based on the existing database must have some deviation from reality. Although these results provided more possibilities and a wider application of this predictive model in a clinical setting, a well-designed and multi-center study is needed for further exploration. Moreover, all mechanical analysis in this study was descriptive, and further, *in vitro* and *in vivo* functional experiments are needed to clarify the underlying mechanism of the eight genes.

In conclusion, by comparing MSI and MSS, we screened significantly different gene expressions and immune cell infiltration. Using WGCNA, we identified eight prognostic signature genes associated with immune infiltration phenotype that presented significant prognostic value. Prognostic and nomogram predictors constructed on the basis of prognostic signatures can stratify and identify the prognostic risk of colon cancer. Furthermore, analyses of immune characteristics revealed a significant positive correlation between *VSIG4* expression and immune cell infiltration, immune score, HLA gene expression, and immune pathways.

## 5 Conclusions

We revealed that eight signature genes related to MSI and immune infiltration in colon cancer showed potential for prognostic risk prediction.
